# Occupational stress profiles of prehospital and clinical staff in emergency medicine—a cross-sectional baseline study

**DOI:** 10.3389/fpubh.2024.1480643

**Published:** 2024-09-30

**Authors:** Christine Meyer, Costanza Chiapponi, Florentin von Kaufmann, Karl-Georg Kanz, Dominik Hinzmann

**Affiliations:** ^1^Chair of Vegetative Anatomy, Institute of Anatomy, Faculty of Medicine, LMU Munich, Munich, Germany; ^2^Department of Surgery, TUM School of Medicine and Health, Munich, Germany; ^3^Department for Command Services and Crisis Management Teams, Munich Fire Brigade, Munich, Germany; ^4^Department of Trauma Surgery, TUM School of Medicine and Health, Munich, Germany; ^5^Department Clinical Medicine, Department of Anesthesiology and Intensive Care Munich, TUM School of Medicine and Health, Munich, Germany

**Keywords:** occupational stress and mental-physical health, social stress and social support, emotional dissonance, emergency nurses, EMS dispatch center, trauma surgeons, emergency medical staff, working condition analysis

## Abstract

**Background:**

Occupational stress among emergency medical staff remains a central problem. Prior to the COVID-19 pandemic, many studies were focused on the working conditions of clinical emergency staff, but few examined the occupational stress profiles of prehospital emergency dispatchers (ED). The aim of this study is therefore to provide baseline data on the differences in occupational stress profiles between prehospital and clinical emergency medical staff.

**Methods:**

ED, emergency nurses (EN), and trauma surgeons on duty (TS) were questioned using the established and validated standardized short version of the instrument for stress-related job analysis for hospital physicians (ISAK-K). Differences between occupational groups were compared using the Mann–Whitney U test.

**Results:**

Our data indicate significant differences in perception of stressors between professional groups (*p* < 0.05), with ED showing the highest psychological stress, followed by EN. Social stressors and emotional dissonance were significantly higher in ED and EN compared to TS (*p* < 0.05). Time pressure was identified as major stressor for ED and TS, but not for EN (*p* < 0.01). All professions showed moderate high levels of uncertainty and frustration (*p* = n.s.). Support from colleagues and supervisors was the greatest positive resource for all professional groups (*p* = n.s.).

**Conclusion:**

In accordance with current literature, our results advocate for a re-evaluation of the identified stressors, as ED, EN, and TS continue to show high levels of occupational stress. Training programs for coping with emotional dissonance and social stressors are likely to be crucial for reducing job stress among ED and EN.

## Graphical abstract

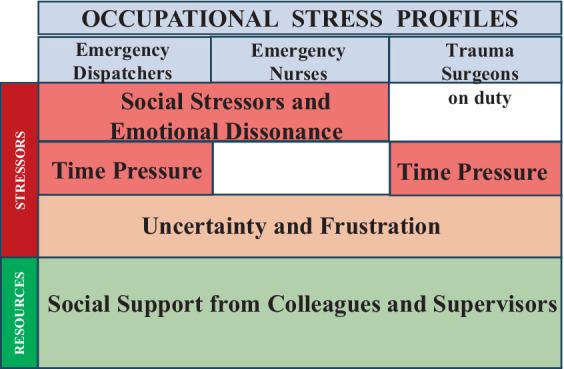

## 1 Introduction

Occupational stress remains a major challenge for emergency medical staff, even after the coronavirus disease (COVID-19) pandemic (1, 2). Although the mental health of medical staff has frequently been at the center of international discourse since the beginning of the pandemic (3), the current cases of illness among nursing staff in Germany reveal an alarming situation.^1^ In view of the upcoming demographic changes in industrialized countries associated with increasing life expectancy, the emergency medical system is facing significant global challenges (4) and, both prehospital and clinical healthcare professionals in emergency medicine need to sustain long-term health and performance.

Measuring quality in emergency medicine is essential because control centers for EMS and emergency departments are High Reliability Organizations (HRO) and they are committed to continuous quality improvement to ensure and improve the quality of care in the long term (5, 6). Quantitative analyses allow rapid assessment of patient numbers, costs and diagnoses, but qualitative values, such as results from staff surveys, are able to provide important indications of promising opportunities for quality improvement (7). However, many qualitative analysis focus on patient satisfaction and its correlation with the quality of care (8), but the well-being of the staff can also be considered as an important indicator for internal quality (9).

The measurement of occupational stress is a multi-faceted interplay of a variety of intrinsic and extrinsic factors, including levels of professional experience, differences in the distribution of roles, levels of patient responsibility and differences in training concepts (10, 11). Also factors such as the level of moral and emotional intelligence as well as personality traits including emotional stability and responsibility have been reported to impact stress levels at work (11, 12). In general, a mismatch between job demands and employees’ ability to cope with daily demands leads to occupational stress, characterized by a complex interplay of several influencing factors (13). According to the transactional stress model of Lazarus and Folkmann, the primary and secondary evaluation of a challenging situation is of crucial importance (14). Primary evaluation refers to the mental evaluation process of the situation by the individual itself, while the secondary evaluation focuses on the resources of the individual, including skills and expertise as well as autonomy at work (14, 15). Both evaluation processes lead to individual coping behavior, which potentially triggers negative stress in the individual if the situation is perceived as particularly stressful or if there is a lack of resources (14, 16).

The limited data available before the pandemic on the stress levels of emergency dispatchers (ED) clearly indicate high levels of occupational stress and increased burnout, highlighting the need for psychological support (17–19), which is also essential considering the link between burnout and the intention to change jobs (20). To the authors’ knowledge, there is no pre-pandemic study comparing occupational stress levels of ED to clinical staff of the emergency department, although all three professional groups work together and ED represent the critical interface between prehospital and clinical emergency medicine (6). Having access to baseline data on job stress due to personal, professional, and organizational weaknesses could support make better decisions about personnel development measures to ensure preparedness for current and future challenges, and can serve as a compelling rationale for internal and political decision makers (16, 21).

Therefore, the aim of this study is to add baseline data on the job stress profiles of ED, EN and TS prior to the COVID-19 pandemic, including job-specific differences, to provide specific approaches to re-evaluate the working conditions of prehospital and clinical staff in emergency medicine.

## 2 Methods

### 2.1 Study design and participants

This cross-sectional survey was originally designed as pilot study, which was conducted before the COVID-19 pandemic. The survey period was from mid-July 2014 to the beginning of August 2014 (department of trauma surgery) and the beginning of October 2016 (control center for EMS Munich). The total sample size across all groups (ED, EN, and TS) was N = 77, who were invited to voluntarily participate in the study.

Inclusion criteria: Specifically, the survey included all EN (*N* = 19) and all TS (*N* = 30) in the department of trauma surgery from a high level I university hospital, representing a complete census of these populations. In contrast, the survey at the control center for EMS Munich was a purposive sample, which was selected by the director and deputy director of the Munich fire brigade control center, resulting in a targeted sample of *N* = 28 ED. ED were between 35 and 58 years old and by the time of the survey they had at least about 3 years of experience in the control center for EMS Munich. Of note, ED profession in Germany is traditionally a male profession, as they work as firefighters in addition to dispatching.

Exclusion criteria: Non-medical staff such as administrative staff and people not directly employed by the trauma surgery department, such as medical staff from other medical departments, medical students, or ED with less than 3 years of work experience in the control center for EMS Munich were excluded from participation.

### 2.2 Study setting and data collection

The invitation for voluntary participation in the written employee survey was communicated to TS during the daily morning meeting and to EN during a separate nursing team meeting, which was also attended by the head of surgical emergency department and the director of the department of trauma surgery who expressed their positive opinion of the project. EN and TS received the paper survey by internal post including an envelope for anonymous return to the secretariat of the department of trauma surgery.

In relation to ED, preselected ED were invited to voluntarily participate in the anonymous paper-based employee survey via e-mail and were informed about the aim and process of the project in a 10-min PowerPoint presentation followed by a Question-and-Answer session. The study was conducted as a paper-and pencil-survey (didactic study design) in a total of four available time blocks before or after the work shift at the control center for EMS Munich.

### 2.3 Survey instrument

The short version of the instrument for stress-related job analysis for hospital physicians (ISAK-K), version 01/2013 (16, 22), from the German social accident insurance (DGUV) for non-state institutions within the health and welfare service sectors (BGW) is a well-established, reliable and valid questionnaire that was originally developed by the BGW for measuring work-related stressors and resources in hospital physicians and is based on the extension of the transactional stress model of Lazarus and Folkman (16, 22). ISAK-K can be downloaded free of charge from the BGW website^2^ and via Leibniz Center for Psychological Information and Documentation (23). Linguistic and contextual modifications were made to accommodate the unique characteristics and requirements of ED (see Supplementary material: Adapted survey questions of the ISAK-K for emergency dispatchers). The questions were closed-ended and could be rated on a 5-point Likert scale from 1 to 5. The survey took approximately 5–10 min to complete. Consent was implied by completing and returning the survey. The 4-page questionnaire ISAK-K consists of 30 items in 14 scales including 7 stressors and 7 resources:

**Table:** 

Stressors	Resources
Social stressors with patients and families/callers Uncertainty Time pressure Emotional dissonance Frustration about how work needs to be done Problems in workflow with other professions Problems in workflow (own professional group or supervisors)	Support from colleagues Support from supervisors Professional and skill development at work Possibilities in further education Autonomy at work Justice (fairness in the distribution of tasks and support) Participation at work

### 2.4 Permission of the staff council

The staff councils of both institutions, the control center for emergency medical services (EMS) Munich and the TUM School of Medicine and Health, Department of Trauma Surgery Munich, have approved the employee survey as part of staff development measures. This approval complies with the requirements of the German Occupational Health and Safety Act (Arbeitsschutzgesetz §5 III Nr. 6)^3^ on psychological burdens in the workplace. Additionally, the department of Human Resources and Organization of the City of Munich approved and accompanied the study at the control center for EMS Munich.

### 2.5 Ethics approval and consent to participate

In accordance with the legal regulations applicable in Germany, no formal ethical approval was required for this study. Employee-related topics such as staff surveys as part of staff development initiatives are approved by the Staff Council (see Permission of the Staff Council), which gave its approval for this study. It should be emphasized, that the questionnaire used in this study was a validated instrument provided by the German social accident insurance (DGUV) for non-state institutions within the health and welfare service sectors (BGW), which was developed specifically for staff development measures for hospital physicians (see section 2.3). All participants were given comprehensive verbal and written information about the project prior to their participation. By voluntarily participating in the survey and submitting their questionnaires, all participants gave their informed consent to take part in the study. To maximize anonymity, no socio-demographic data were collected in this study.

### 2.6 Data analysis

Data analysis was performed by downloading the ISAK-K analysis program free of charge from the BGW website.^4^ Surveys with incomplete answers or multiple answers to a question were excluded due to limitations of the ISAK-K software. The data set was extracted using Excel and the descriptive data including median and interquartile ranges were calculated after transferring the data to IBM SPSS for Windows 2000. Differences between professions were analyzed using the Mann–Whitney U test, whereby the significance level α was set at 5%. The standard scatter range of the BGW was based on the range of the ISAK-K reference study (16) and median values were kindly provided by the authors of the original ISAK-K development (22). Box plots were generated with the statistical program R and PowerPoint for Mac OS 2021.

## 3 Results

A total of *N* = 59 questionnaires were completed and returned by employees (*N* = 28 ED, *N* = 13 EN, and *N* = 18 TS), resulting in an average response rate of 76.7%. In the ED group, 5 out of the 28 returned questionnaires (18%) were excluded from the analysis due to incomplete responses, as the BGW-ISAK-K software requires complete responses for each scale. Consequently, N = 54 questionnaires (*N* = 23 ED, *N* = 13 EN, *N* = 18 TS) were included in the final analysis.

**Figure 1:**
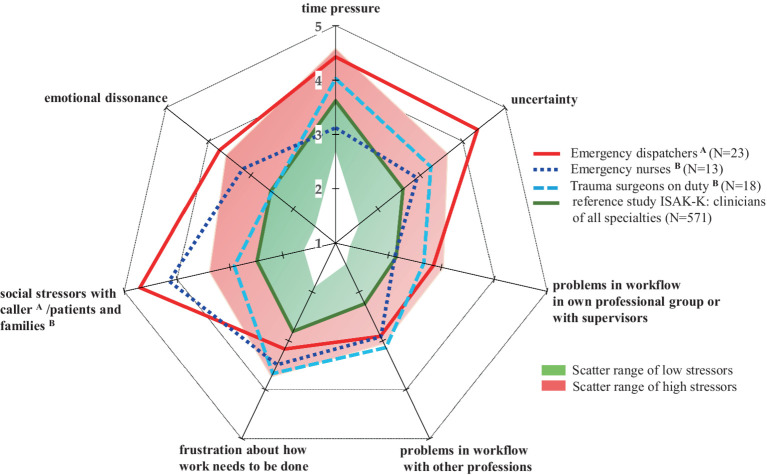
Stress profiles of emergency dispatchers, emergency nurses and trauma surgeons. Data are shown as mean values on a 5-point Likert scale from 1 to 5. Each value corresponds to the subjectively perceived level of stressor (“1” = low stressor; “5” = high stressor). The scatter range was defined in the ISAK-K software based on a reference study with hospital physicians. ISAK-K, short version of the instrument for stress-related job analysis for hospital physicians; *N*, number of participants.

Compared to the scatter range of the ISAK-K reference study, all professions showed increased stress profiles, with ED showing the highest values, followed by EN (Figure 1). Social stress from callers, emotional dissonance, uncertainty about how work needs to be done and time pressure were the greatest source of stress for ED (Figure 1). Social stressors with patients were also a major stress factor for EN, combined with emotional dissonance, frustration and problems with workflow, while time pressure was in the scatter range of low stressors (Figure 1). TS showed high causes of stress relating to time pressure, uncertainty, and frustration, while social stress factors from the patients also showed a medium stress factor, but with a low level of emotional dissonance (Figure 1). The t-test revealed a non-normal distribution of the survey results in all three populations.

### 3.1 Social stressors and emotional dissonance

**Figure 2:**
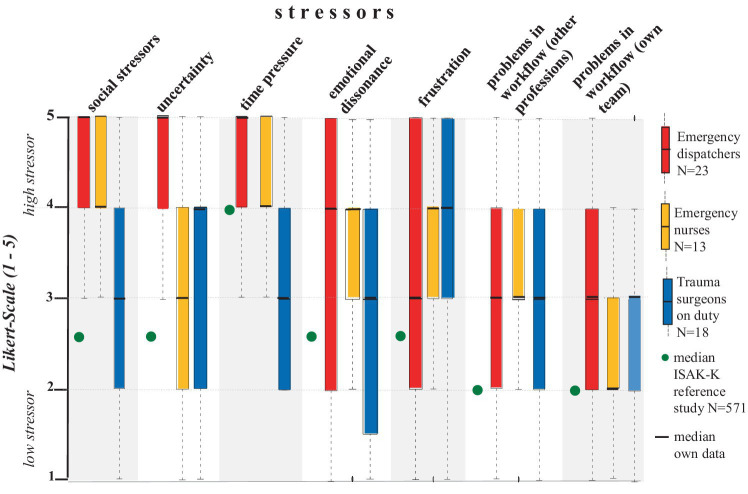
Distribution of stressors as boxplots for emergency dispatchers, emergency nurses and trauma surgeons. Data are shown on a 5-point Likert scale from 1–5. Each value corresponds to the subjectively perceived level of stressor (“1” = low stressor; “5” = high stressor).

The comparison between professions showed significant differences between ED, EN and TS regarding the evaluation of major stressors (*p* < 0.05) (Figure 2; Table 1). ED and EN reported significantly higher perceptions of stress compared to TS for social stressors and emotional dissonance (*p* < 0.05) (Figure 2; Table 1). At least 50% of ED (med = 4.0; IQR = 3) and at least 50% of EN (med = 4.0; IQR = 1) reported having to suppress their feelings ‘almost every day’ to ‘several times a day’ (Figure 2). The comparison between ED and EN further showed that ED rated social stressors significantly higher than nurses (*p* = 0.18), whereas there was no significant difference in emotional dissonance (*p* = n.s.; Table 1). TS rated the emotional dissonance factor significantly lower than ED (*p* = 0.003) and EN (*p* = 0.046) (Table 1).

**Table 1:** Comparison of job stressors and resources among emergency dispatchers, emergency nurses and trauma surgeons.

	Profession				Differences		
	ED	EN	TS	Benchmark-Data ISAK-K	ED vs. EN	ED vs. TS	EN vs. TS
	*N* = 23	*N* = 13	*N* = 18	*N* = 571			
Stressors	Med (IQR)			Med	*p*-value^*^		
Social stressors with patients and families/callers	5.0 (1)	4.0 (1)	3.0 (2)	2.5	**0.018**	**0.000**	**0.000**
Uncertainty	5.0 (1)	3.0 (2)	4.0 (2)	2.5	**0.000**	**0.000**	0.588
Time pressure	5.0 (1)	3.0 (2)	4.0 (1)	4.0	**0.000**	**0.011**	0.**002**
Emotional dissonance	4.0 (3)	4.0 (1)	3.0 (2.5)	2.5	0.154	**0.003**	**0.046**
Frustration about how work needs to be done	3.0 (3)	4.0 (1)	4.0 (2)	2.67	0.291	0.069	0.297
Problems in workflow with other professions	3.0 (2)	3.0 (1)	3.0 (2)	2.0	0.726	0.452	0.730
Problems in workflow (own team)	3.0 (2)	2.0 (1)	3.0 (1)	2.0	0.217	0.634	0.409
Resources							
Support from colleagues	4.0 (1)	4 (1)	4.0 (1)	4.0	0.597	0.519	0.889
Support from supervisors	3.5 (1)	3 (1)	4.0 (2)	3.5	0.693	0.298	0.257
Professional and skill development at work	3.0 (2)	4 (1)	4.0 (2)	3.0	0.051	0.768	**0.049**
Possibilities in further education	4.0 (1)	3 (1)	3.0 (2)	3.5	0.066	0.180	0.805
Autonomy at work	4.0 (2)	3 (1)	3.0 (2)	3.5	**0.000**	**0.002**	0.414
Justice (distribution of tasks and support)	3.0 (1)	3 (1)	3.0 (2)	3.0	**0.015**	0.742	0.070
Participation at work	2.0 (2)	3 (1)	3.0 (1.5)	2.5	0.130	0.106	0.894

Mann–Whitney U test was applied for statistical analysis. The significance level α was set at 5 percent. Data are shown based on 5-point Likert scale from 1–5. Each value corresponds to the subjectively perceived level of stressor (“1” = low stressor; “5” = high stressor) or resource (“1” = low resource; “5” = high resource). ED, emergency dispatchers; EN, emergency nurses; IQR, interquartile range; ISAK-K, short version of the instrument for stress-related job analysis for hospital physicians; med, median; TS, trauma surgeons on duty.

* Significant *p*-values are bold.

### 3.2 Time pressure, frustration, and uncertainty in decision-making during work

ED, EN and TS all indicated a tendency for uncertainty and frustration at high stressor levels, whereas time pressure has been found as major stressor among ED and TS but not EN. ED rated uncertainty in decision-making during work significantly higher than EN (*p* = 0.000) and TS (*p* = 0.000; Table 1). The responses of both EN and TS showed an overall wider range (*p* = n.s.; Figure 2; Table 1). ED and TS reported significantly higher affectedness than EN for time pressure (*p* < 0.01; Figure 2; Table 1). The comparison between ED and TS further showed that ED rated time pressure significantly higher than TS (*p* = 0.011). All occupations showed a trend towards a moderately high level of frustration (*p* = n.s.; Figure 2; Table 1).

### 3.3 Social support at work as the greatest resource at work

**Figure 3:**
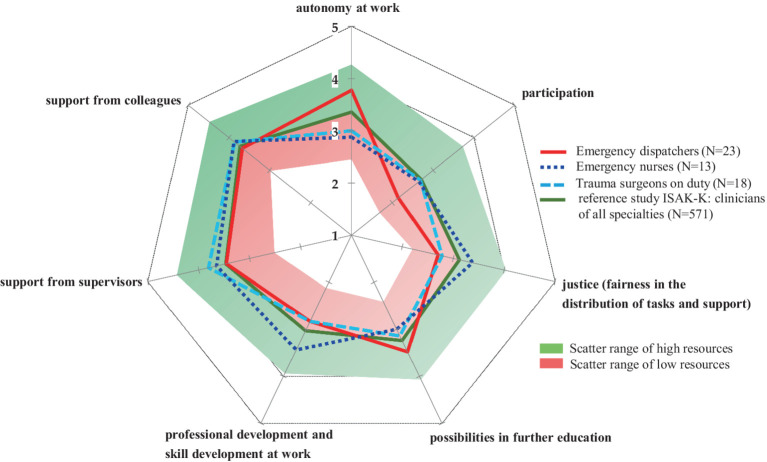
Resource profiles of emergency dispatchers, emergency nurses and trauma surgeons. Data are shown as mean values on a 5-point Likert scale from 1–5. Each value corresponds to the subjectively perceived level resource (“1” = low resource; “5” = high resource). The scatter range was defined in the ISAK-K software based on a reference study with hospital physicians. ISAK-K, short version of the instrument for stress-related job analysis for hospital physicians; *N*, number of participants.

Overall, all occupations showed a broadly balanced resource profile, with four out of seven resources in the high scatter or benchmark range (Figure 3). This is an important result, especially with the high stressor profiles found among ED as described in the last section. In addition, ED rated autonomy at work and possibilities in further education within the scatter range of high resources (Figure 3). However, the results clearly showed a trend towards optimizing and strengthening specific work resources, such as work participation for all professional groups, and individual factors, such as strengthening work autonomy for emergency clinical staff (Figure 3).

**Figure 4:**
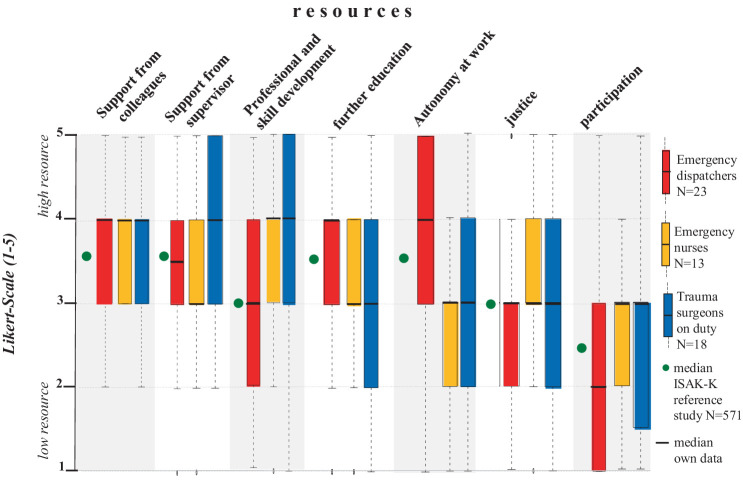
Distribution of resources as boxplots for emergency dispatchers, emergency nurses and trauma surgeons. Data are shown on a 5-point Likert scale from 1–5. Each value corresponds to the subjectively perceived level of resource (“1” = low resource; “5” = high resource).

Notably, ED, EN and TS all reported support from colleagues and superiors as their greatest positive resource (*p* = n.s.) and participation as their lowest resource (*p* = n.s., Table 1). The factor social support from colleagues in their own professional group was rated in the high resource range by ED (med = 4.0; IQR = 1), EN (med = 4.0; IQR = 1) and TS (med = 4.0; IQR = 1; Figure 4). At least 50% of each occupational group reported that they could rely ‘fairly’ to ‘completely’ on their team colleagues when things got difficult at work, and at least 50% of TS also reported that they could rely ‘fairly’ to ‘completely’ on their supervisors when things got difficult at work without group differences (med = 4.0; IQR = 2, Figure 4). With regard to autonomy and decision making, ED rated this factor as a higher resource than EN (*p* = 0.000) and TS (*p* = 0.002), but it should be noted that this factor is not directly comparable due to linguistic modifications of the survey question in relation to the working conditions among ED (see Methods section). Fairness in workplace task allocation was rated as a higher resource by TS than by ED (*p* = 0.015) but no significant difference was found between EN and TS or between EN and ED (Table 1).

## 4 Discussion

Overall, taking into account the stressor and resource profiles specific to each occupational group, our pre-pandemic data supports the findings of the March–October 2020 COVISTRESS international study, which found that prehospital staff are exposed to higher levels of occupational stress than clinical staff in emergency medicine (24). This is in contrast to other data showing a lower burden of stress on prehospital compared to clinical emergency staff (25). Nevertheless, there is a clear trend that stress levels remain high among both prehospital and clinical emergency staff when the occupational groups are considered separately (26–28), especially due to the fact that actual stress profiles are likely to be greater than the sum of the individual stressors due to cumulative synergistic effects (29). Hence, there is a clear need to further strengthen resources, including a high level of support from the team and superiors, based on a sustainable organizational philosophy (30, 31). In view of the fact that subjective and objective data often coincide when measuring chronic stress in individuals (19), both types of data including those from our study, will be discussed accordingly in the following section.

### 4.1 Social stressors and emotional dissonance

Pre-pandemic studies identified constant high-complexity incoming calls as a significant stressor among ED with increased salivary cortisol levels (32, 33). The daily total cortisol concentrations among ED correlated with subjective perceptions of emotional distress (34) and other objective data such as elevated heart rate among ED have also been reported (18). Both chronically elevated cortisol levels and elevated heart rate are considered biomarkers of chronic stress with a disrupted hypothalamic–pituitary–adrenal axis and an increase in pro-inflammatory cytokines (35), which affect an individual’s epigenetics and correlate with many chronic inflammatory diseases (36). Subjectively perceived stressors therefore often reflect stress biomarkers (37), which is consistent with Lazarus and Folkmann’s transactional stress model, which describes that the primary and secondary evaluation of a difficult situation is crucial (14). Our data consequently indicate that ED have difficulty of coping with negative emotions (38) reflecting earlier pre-pandemic findings identifying communication issues with difficult callers among ED (39). Similarly, EN appear to face a comparable constant burden when dealing with difficult patients and their relatives in the emergency department, both before (40) and after the COVID-19 pandemic (41), in line with our findings. Pre-pandemic data demonstrate that 46% of EN actively experience physical aggression from intoxicated patients, compared to only about 20% of physicians (42), explaining the differences in social stressors between the nursing and physician professions. A retrospective study of a German high level I university hospital confirmed that violence in emergency departments occurs approximately every 0.7 days (43). Another aspect is that emergency physicians face a lower number of daily patient contacts and a higher proportion of indirect patient work compared to EN who have a higher percentage of direct patient work time (44–46). Moreover, it has been reported that the amount of patient communication correlates with the level of social stressors (22). Based on data of the German Trade Union Confederation (DGB) “Good Work Index,” the level of external interaction work further correlates with the level of emotional dissonance, especially in health care professions^5^ (47). Emotional dissonance indicates the conflict between the emotions experienced and the emotions expressed in order to fulfill the required external representation of the professional role (48). According to Ellis’ concept of Rational Emotive Behavior Therapy (REBT) the ability for rational thinking is a key factor for handling social stressors and emotional dissonance effectively (49). This concept has already been shown as an effective intervention for reducing irrational performance beliefs in firefighters (50). Most physicians working in a high-level I university hospital are typically involved in publishing scientific papers on a regular basis and are therefore trained in scientific thinking, which may partly explain why they experience less emotional dissonance. In view of the fact, that emotional dissonance correlates with irritability and emotional exhaustion (22) and has been shown to be a stronger indicator of burnout than time pressure (51), the high levels of emotional dissonance among ED and EN suggest a specific call to action, such as implementing training modules to manage negative emotions, including concepts such as REBT (50, 52) or the Nonviolent Communication model of Marshall Rosenberg (53). Appropriate training modules in emotion regulation, resilience, and health education were already shown to reduce nurses’ workplace stress levels (52, 54, 55).

### 4.2 Time pressure, frustration and uncertainty in decision-making during work

Time pressure has been reported as major stressor among German hospital physicians (15, 22, 56), independent from specialties (22) and age (56), indicating to a general problem such as the high documentation burden (57). Time pressure positively correlates with frustration about how work needs to be done including too much documentation (22), which is in line with our findings. Interestingly, surgeons have been found with the highest work ability index and the lowest depression scores compared to other specialties, even though they are exposed to a high level of stress, such as time pressure (58), indicating high psychological resilience. EN did not rank time pressure as a major stressor, which is consistent with other research data (41). Nurses in health care systems like Germany have less responsibility and decision-making authority, which might correlate with less time pressure (59). An approach to reduce time pressure is to focus on reducing frustration first through specific training of key qualifications for handling administrative work and also on organization level including a reduction in documentation and clear instructions such as standard operating procedures (22). However, the most useful intervention to reduce physician stress appears to be the further development of the framework and range of non-patient tasks, such as documentation, using technological tools such as artificial intelligence (22, 60).

In terms of uncertainty at work, data during the COVID-19 pandemic showed that nurses had significantly higher levels of anxiety, depression and general fear compared to physicians and paramedics, combined with lower levels of subjective information, suggesting that regular educational training has the potential to improve confidence and counteract uncertainty by providing sufficient background knowledge, particularly for EN (61). Insufficient information from physicians about the patient’s medical condition has been reported of being the root cause of nurses’ uncertainty (41). A high level of uncertainty among ED with regard to emergency calls has been shown to correlate with lack of guidance, feedback and training (39), supporting the valuable factor of encouraging and productive feedback as leadership tool (31) which was recently reported in EMS staff associated with improved clinical performance (62). Internal managers and policy makers should be aware that if stress profiles remain high, intentions to leave will increase (20).

### 4.3 Social support at work

According to Johnson and Hall’s extension of Karasek’s job-demand-control model (63), a high level of social support acts as a positive modulating factor for occupational stress and chronic diseases (64). Indeed, social support at work and mindfulness correlated with stress levels among ED (65). Data from the German Stress Report 2019 have shown that 80% of employees in healthcare professions rate social support from colleagues as frequent and social support from supervisors as more than 50% frequent, which has remained consistent over the past few years (66), in line with our findings. This fact is particularly remarkable as other studies have found low team support among emergency medicine staff (67, 68). Moreover, since our data indicate a positively strong resource in terms of both colleagues and supervisors compared to other data (16, 56), it is reasonable to assume a positive, exemplary leadership culture correlates with improved team support, which is an essential factor particularly in HRO such as the emergency department and EMS control center (5).

## 5 Limitations

This study was originally designed as a pilot study to identify weaknesses in prehospital and clinical emergency medical staff and to re-evaluate the data in follow-up studies. Due to limited personnel resources as well as the development of the global COVID-19 pandemic, we were unable to realize further follow-up studies and our data therefore cannot be generalized to the current post-pandemic working conditions. Therefore, the comparison presented in the discussion between data on work-related stress collected during and after the pandemic must be interpreted with caution. Besides the temporal differences, the data come from different institutions with varying workplace conditions, so the comparison serves only as a general overview. In addition to the impact of the pandemic, other factors such as generational shifts within the healthcare system have also intensified, potentially affecting stress levels differently than in our study (69, 70).

The results are subject to selection bias due to the voluntary nature of participation, and the cross-sectional design of the study does not allow causal conclusions to be drawn. The validity and reliability of the ISAK-K does not apply to the results of ED and EN, as the original ISAK-K questionnaire survey was developed exclusively for hospital physicians, which also applies to the ISAK-K benchmarking data (16). Some scales are not directly comparable between the ED, EN, and TS due to linguistic modifications. A further limitation results from the different responsibilities and workplace conditions between the professional groups, which for ED is a computer workstation without direct patient contact, whereas EN and TS have direct patient contact. In addition, differences in socio-economic status between ED, EN and TS are very likely. Gender differences may also be limiting, as there is still a male predominance in the ED and TS profession in Germany, while there is still a female predominance in the EN profession. The ISAK-K guidelines recommend a minimum of N = 7 participants for the evaluation of the ISAK-K to ensure adequate anonymity, and this requirement was met for all groups in our study. However, it is important to note that the relatively small sample size may impact the statistical significance and generalizability of the findings. Despite this limitation, we observed significant differences between the groups, suggesting meaningful variations between occupational groups. We recommend that future studies with larger sample sizes be conducted to validate the robustness and generalizability of these results. In addition, the exclusion of 5 questionnaires (18%) from the ED group may have introduced some bias, potentially affecting the representativeness of the results for this subgroup. However, no imputation methods were applied, as the missing data were considered random, with no indication of systematic bias.

The differences in the timing of data collection between the control center for EMS Munich and the department of trauma surgery resulted from different administrative processes and approval requirements. In particular, the process for the control center for EMS Munich required more extensive planning and formal approval due to its affiliation with the Munich Fire Department and, consequently, the City of Munich. These differences highlight practical considerations for future research in similar settings. In this context, it is important to note that data collection at the control center for EMS Munich unintentionally occurred immediately after the 2016 Munich Oktoberfest. This timing may have introduced additional stressors due to increased call volume and emergency events associated with the festival, potentially leading to elevated stress levels among ED staff (71, 72). Consequently, a follow-up study conducted during a less stressful period is recommended to enable a more accurate comparison.

## 6 Conclusion

This cross-sectional study showed that pre-hospital and clinical emergency staff had higher stress profiles than external benchmark data for hospital physicians of different specialties. Internal data from the emergency department and the EMS control centre show that emergency dispatchers (ED) were exposed to the highest occupational stress profiles before the COVID-19 pandemic, mainly due to social stressors and emotional dissonance, followed by emergency nurses (EN), while trauma surgeons were significantly less affected. Therefore, our study advocates for a re-evaluation of the psychological risk assessment, particularly for ED and EN. The study data also advocate integrating these baseline data into policy considerations to optimally prepare for future challenges, including demographic changes that may place additional strain on the emergency medical system, requiring proactive decisions to sustain the overall health of prehospital and clinical emergency staff.

## Data Availability

The original contributions presented in the study are included in the article/Supplementary material, further inquiries can be directed to the corresponding author.

## Footnotes

^1^Available from: https://www.tk.de/presse/themen/pflege/pflegepolitik/krankenstand-bei-pflegekraeften-auf-rekordhoch-2149302 last accessed on 24.06.2024.

^2^Available from: https://www.bgw-online.de/bgw-online-de/service/medien-arbeitshilfen/medien-center/stressbezogene-arbeitsanalyse-14858 last accessed on 13.08.2024.

^3^Available from: https://www.gesetze-im-internet.de/arbschg/__5.html last accessed on 13.08.2024.

^4^Available from: https://www.bgw-online.de/resource/blob/9114/673a348e7124869c9886353fb2d17318/auswertung-data.bin last accessed on 13.08.2024.

^5^Available from: https://index-gute-arbeit.dgb.de/++co++60787eb2-6b37-11ed-8d14-001a4a160123 last accessed on 13.08.2024.
