# Correction: Occupational stress profiles of prehospital and clinical staff in emergency medicine—a cross-sectional baseline study

**DOI:** 10.3389/fpubh.2026.1803339

**Published:** 2026-02-26

**Authors:** Christine Meyer, Costanza Chiapponi, Florentin von Kaufmann, Karl-Georg Kanz, Dominik Hinzmann

**Affiliations:** 1 Chair of Vegetative Anatomy, Institute of Anatomy, Faculty of Medicine, LMU Munich, Munich, Germany; 2 Department of Surgery, TUM School of Medicine and Health, Munich, Germany; 3 Department for Command Services and Crisis Management Teams, Munich Fire Brigade, Munich, Germany; 4 Department of Trauma Surgery, TUM School of Medicine and Health, Munich, Germany; 5 Department Clinical Medicine, Department of Anesthesiology and Intensive Care Munich, TUM School of Medicine and Health, Munich, Germany

**Keywords:** occupational stress and mental-physical health, social stress and social support, emotional dissonance, emergency nurses, EMS dispatch center, trauma surgeons, emergency medical staff, working condition analysis

There was a mistake in Figure 2 as published. In the boxplots illustrating the stressor *time pressure*, the occupational groups *emergency nurses* and *trauma surgeons on duty* were inadvertently interchanged in the graphical representation. The corrected Figure 2 appears below. This correction does not affect the underlying data, analyses, or scientific conclusions of the article.

**Figure 2:**
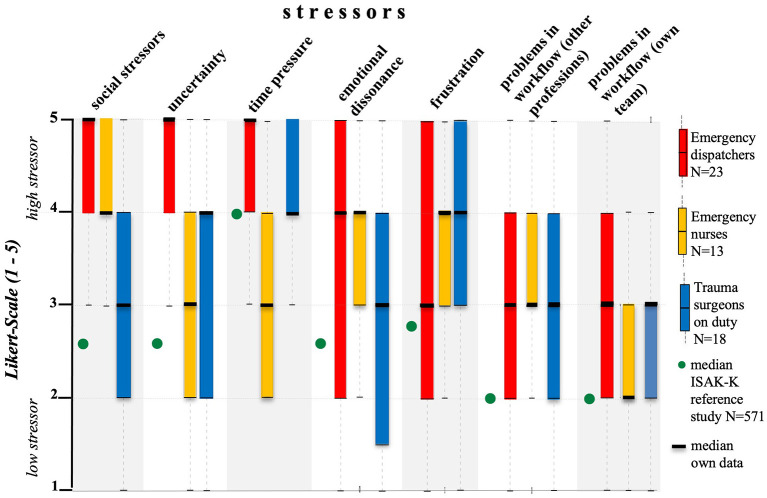
Distribution of stressors as boxplots for emergency dispatchers, emergency nurses and trauma surgeons. Data are shown on a 5-point Likert scale from 1 to 5. Each value corresponds to the subjectively perceived level of stressor (“1” = low stressor; “5” = high stressor).

The original version of this article has been updated.

