# Supplementary material for: Occupational stress profiles of prehospital and clinical staff in emergency medicine—a cross-sectional baseline study
Source: Front Public Health. 2024 Sep 30;12:1480643. doi: 10.3389/fpubh.2024.1480643 (PMC11472573; doi:10.3389/fpubh.2024.1480643)
Supplement: Supplementary file 1 [file Data_Sheet_1.docx]

Supplementary Material

# Adapted survey questions of the short version of the instrument for stress-related job analysis for hospital physicians (ISAK-K) (1) for emergency dispatchers.

**Scale: Time Pressure**

Q (Question) 1: How often are you under time pressure?

Q 2: How often do you have to make important decisions under time pressure (e.g., 'collapse vs. unconsciousness')?

**Scale: Uncertainty**

Q 3: How often do you have to make decisions without having sufficient information?

Q 4: How often do you have to make decisions where you find it difficult to assess the consequences?

**Scale: Frustration**

Q 5: How often does it happen that the time for an emergency call is too short because there are too many calls on hold?

Q 6: How often do you conduct an emergency call differently than you consider appropriate due to guidelines from the clinic and/or superiors?

Q 7: In terms of your own standards for your work, how often do you find yourself in a conflict between legal requirements and the caller's/family member's interests/concerns (e.g., patient information about whereabouts)?

**Scale: Autonomy and Decision-Making Authority**

Q 8: How much freedom do you have to determine how you carry out your work?

Q 9 How much freedom do you have to determine how you spend your on-call time in terms of sports, massages, TV, and the internet?

**Scale: Opportunities for Development at Work**

Q 10 How much opportunity do you have to learn new things in your work?

Q 11 How much variety does your work offer you?

**Scales: Collaboration with Colleagues and Superiors and Collaboration with Other Professional Groups**

How often do these individuals provide you with information/documents late, not at all, or incorrectly?

Q 12 Shift group leader + Teammates regarding the CRM guideline "communicate safely and effectively"

Q 13 Colleagues from the emergency medical services

How often do these individuals fail to adhere to agreements with you?

Q 14 Shift group leader + Teammates regarding the CRM guideline "communicate safely and effectively"

Q 15 Colleagues from the emergency medical services

**Scale: Social Stressors**

The following questions relate to your work with callers and family members

Q 16 How often do callers or family members make excessive demands on you?

Q 17 How often do callers or family members blame you?

**Scale: Emotional Dissonance**

Q 18 How often do you have to suppress your own feelings to appear "neutral" outwardly in your work?

Q 19 How often do you have to display emotions outwardly that do not match your true feelings?

**Scales: Social Support from Colleagues and Social Support from Superiors**

The following questions are about your superiors and colleagues:

To what extent can you rely on the following individuals when work becomes difficult?

Q 20 Your direct shift group leader

Q 21 Teammates

How much do these individuals support you to make your work easier?

Q 22 Your direct shift group leader

Q 23 Teammates

**Scale: Participation**

Q 24 I have sufficient influence on the design of work processes that directly affect me.

Q 25 I have sufficient influence on the design of changes within the workplace.

**Scale: Opportunities for Further Education and Training**

Q 26 In our department, inexperienced colleagues have sufficient opportunity to benefit from the knowledge and skills of experienced colleagues.

Q 27 The training of dispatchers is well promoted in our department. (Training = becoming a dispatcher)

Q 28 The professional development of dispatchers is well promoted in our department. (Professional development = becoming a better dispatcher)

**Scale: Justice**

Q 29 When comparing all dispatchers in our department, the shift scheduling is fair.

Q 30 When comparing all dispatchers in our department, the allocation of particularly popular and unpopular tasks is fairly distributed.

**Abbreviations**

*ISAK-K* - short version of the instrument for stress-related job analysis for hospital physicians

*Q*  - Question
